# Design and development of high clearance e-vehicle for robotic cotton picker

**DOI:** 10.1038/s41598-026-50263-1

**Published:** 2026-06-11

**Authors:** Rahul Yadav, Manjeet Singh, Aseem Verma, Derminder Singh

**Affiliations:** 1 Department of Farm Machinery and Power Engineering, Punjab Agricultural University, Ludhiana, 141004 Punjab India; 2 College of Agricultural Engineering & Technology, Punjab Agricultural University, Ludhiana, 141004 Punjab India; 3 Department of Electrical Engineering and Information Technology, Punjab Agricultural University, Ludhiana, 141004 Punjab India

**Keywords:** Centre of gravity, Cotton fields, Downgrade slope, E-vehicle, Moment of inertia, Permanent magnet synchronous motor, Electrical and electronic engineering, Mechanical engineering, Renewable energy

## Abstract

The development of high-clearance, electrically powered agricultural machinery is essential for improving farming efficiency and sustainability. This study presents the design and development of a high-clearance e-vehicle to support a robotic cotton picker, enabling efficient navigation through densely planted cotton fields. A Python-based computational model was used to determine significant vehicle parameters, including centre of gravity, slope stability, turning dynamics, and moment of inertia. The centre of gravity was located at 585.54 mm longitudinally, 867.47 mm laterally, and 1083.46 mm in height from reference points. Stability analysis revealed maximum upgrade and downgrade slopes of 28.39° and 43.67°, respectively, with critical turning speeds of 24.71 km/h for left turns and 27.26 km/h for right turns. The moment of inertia about the centre of gravity was calculated as 51135.31 kg-mm-s^2^. Additionally, the vehicle’s speed performance was evaluated under different motor speeds and gear settings, with a maximum speed of 12.19 km/h achieved in motor speed mode 1 at gear 2. The results confirm that the e-vehicle maintains stability while manoeuvring slopes and turns, effectively operating in fields with row spacings of 900 mm and 675 mm. This study contributes to the advancement of energy-efficient agricultural machinery, addressing challenges in navigating complex crop geometries and supporting the transition to sustainable precision farming.

## Introduction

Cotton is an important crop worldwide, often referred to as “white gold”^1^. In 2024–2025, global cotton production is estimated at 26.1 million metric tonnes, with India contributing about 5.2 million metric tonnes^2^. In cotton fields, the plants are closely spaced, leaving only narrow paths for machines to move through^3^. Cotton-picking robots face tough conditions, where their ability to move and steer is key to reaching their targets. These robots can use different movement methods like legs, wheels, wings, or propellers^4^. Maja et al.^5^ created a mobile platform for fieldwork that is 68 cm wide, designed to fit standard cotton rows. It can carry up to 75 kg and travel at a maximum speed of 1 m/s. The platform runs on two 24-volt lithium polymer batteries, each with 6 cells and a 10 Ah capacity, giving it a runtime of about 3 hours.

Fue et al.^6^ created a cotton-picking robot called the Rover, which was 340 cm long. The front part was 145 cm, and the back part was 195 cm. It was 212 cm wide, and each tire was 30 cm wide with a radius of 30.48 cm and a circumference of 191.51 cm. The front and rear axles were 91 cm from the centre, and it had a ground clearance of 91 cm. Two servos controlled the hydraulic motors for the wheels-one for engine speed and one for the pump. The engine, a Kohler Command 20 hp, ran at up to 2500 rpm and powered a pump that could move 14.1 cubic centimetres of fluid per turn. The Rover could move forward, backward, and turn. Thapa et al.^7^ built a small red rover with four-wheel electric steering. It measured 123 cm in length, 76 cm in width, and 116 cm in height, and it moved between crop rows. Each wheel used a 250 W wheelchair motor, and the front wheels were steered with a linear servo. Cai et al.^8^ created a plant protection machine with adjustable speed and power. It was 2200×1400×1800 mm in size, had 980 mm of ground clearance, and could travel at speeds between 0 and 10 km/h. In the field, it was powered by a diesel engine.

Agriculture is a major source of global greenhouse gas emissions, accounting for over 20% of carbon dioxide equivalents, 42% of methane, and 75% of nitrogen oxides^9^. Diesel-powered farm machinery contributes significantly to environmental pollution by releasing harmful gases into the atmosphere. Diesel engines have many moving parts, and the way they burn fuel causes a lot of noise and vibrations that can hurt the health of workers^10^. Switching to electric-powered machines and using renewable energy is important for improving modern farming. Electric vehicles help reduce the need for fossil fuels, lowering noise and air pollution^11^. They also improve the quality of work and make it more comfortable for operators than traditional fuel-powered machines^12^. In a cotton field, a mobile vehicle plays a crucial role, as it has to carry all other systems and move within the specified geometry of the cotton crop. Also, a big challenge to running a vehicle in the busy cotton field. Considering these challenges, the present study aims to develop an electrically powered high-clearance vehicle suitable for robotic cotton harvesting in narrow-row geometries.

### Characteristics of cotton crop

Kavya et al.^13^ conducted a field trial on Bt cotton NCS-2778 BG-II using a high-density planting system. Bt cotton has substantially improved yields and enhanced the economic outcomes for cotton farmers^14^. They observed that the tallest plants (102.8 cm) were at a spacing of 90 x 15 cm, while the shortest plants (94.2 cm) were at a spacing of 90 x 60 cm. The plants grew taller in crowded conditions because of competition for resources. Sui et al.^15^ found that the average plant height in irrigated plots was 25.0 cm, while in non-irrigated plots, it was 95.1 cm. Yadav et al.^16^ found that the cotton plants had a height of 117.4 cm, which aided in refining the design of the cotton uprooter and shredder machine. Goyal et al.^17^ studied different types of cotton to see if they worked well with modern cotton-picking machines. They discovered that the plants were between 50 and 120 cm tall. On average, the plant canopy was 65 cm wide along the row and 60 cm wide across the row, with 90 cm between plants and 67.5 cm between rows.

**Fig. 1:**
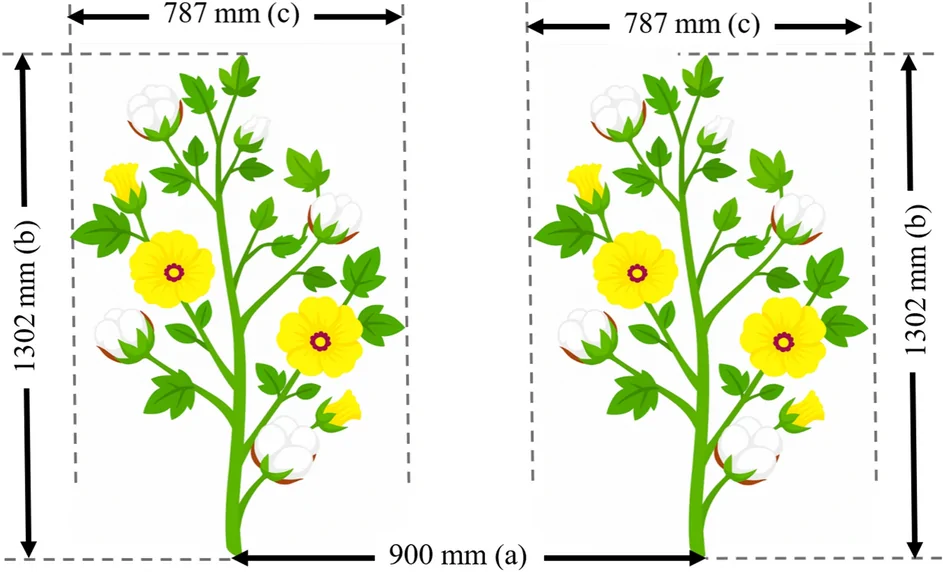
Cotton plant geometry row to row spacing (**a**), plant height (**b**), and plant canopy (**c**).

The Bt hybrid cotton variety RCH 846 BG II was sown using a High-Density Planting System (HDPS) at the research farm of the Department of Farm Machinery and Power Engineering, Punjab Agricultural University in Ludhiana. The row-to-row spacing of the crop was 900 mm. The maximum plant height and plant canopy across row are shown in Fig. 1.

The review of cotton plant height was essential for ensuring vehicle ground clearance, while the plant canopy height and row-to-row plant spacing were important for determining the track width of the vehicle.

### Design constraints and performance targets for the high-clearance e-vehicle

Before undertaking the detailed mechanical design and stability analysis, a set of clearly defined design requirements and operational performance targets was established to ensure that the high-clearance e-vehicle satisfies both agronomic field conditions and operational constraints. These requirements are summarized in Table 1.

**Table 1:** Design constraints and operational targets for the high-clearance e-vehicle.

Parameter	Targets	Source	Justification
Ground clearance	1320 mm	Cotton plant height typically ranges from 500 to 1200 mm^17^,Bt hybrid cotton varieties have reported heights up to 1302 mm	To enable vehicle operation over mature cotton crops with minimal crop damage
Track width	1800 mm	High-density planting system (HDPS) with row-to-row spacing of 900 mm^14^	Allows two cotton rows within the track width while maintaining vehicle stability and an appropriate height-to-width ratio
Wheel width	100 mm	Use of puncture-free, non-pneumatic tires suitable for agricultural applications^8^	Narrow wheel width facilitates movement through dense crop rows while reducing soil and crop disturbance.
Vehicle speed	≤ 12 km/h	Typical agricultural field vehicle speeds range between 0 and 10 km/h^8^	Ensures safe navigation, stability, and precise operation during robotic cotton picking
Operating time	3–4.5 h	Robotic cotton picking involves intermittent movement between plants rather than continuous travel^7^	Provides sufficient operational duration to complete an 8 h field task through task-based vehicle operation

### Chassis, front axle and steering

The e-vehicle chassis was made of strong 80×40×4 mm mild steel, weighing 6.5 kg per meter. Mild steel was selected due to its easy availability, machinability, and weldability. A basic grade of mild steel was chosen for the frame, providing adequate strength against various possible stresses^18^. Its overall size was 3050×1200×80 mm, giving space for motor, axles, arms, and batteries. The front axle beam measures 1240 mm, with a 770 mm hollow cylinder. The steering system used a rack and pinion setup to turn the front wheels, enabling the driver to manually control the direction of the e-vehicle^19^. When the steering wheel turns, a small gear moves a rack, which shifts rods to steer the wheels.

### Design of gearbox and axle shaft

**Fig. 2:**
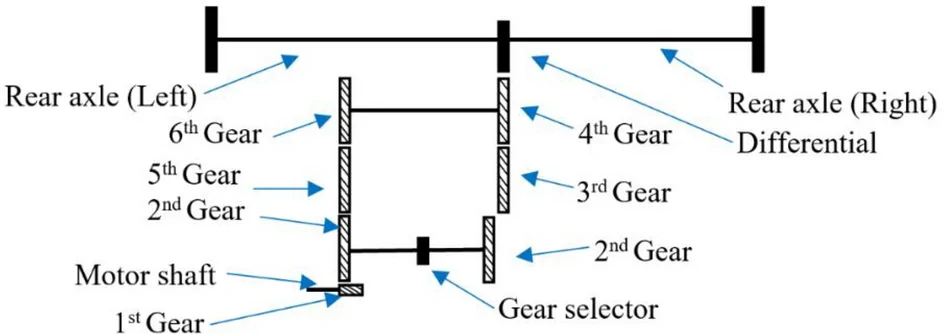
Gearbox.

The gearbox is a component that contains a gear train, which can alter speed, increase torque, and facilitate power transmission from the motor to the axle shaft. The sliding mesh gearbox provides durability in low-speed applications, ease of integration, and relatively lower weight^20^. A two-speed sliding mesh gearbox was designed as shown in Fig. 2. The maximum output required from the gearbox was 242 rpm at 1^st^ speed (*N*_*6*_) and 478 rpm at 2^nd^ speed (*N*_*4*_). Considering that the transmission between the input shaft (*N*_*i*_)and intermediate shaft (*N*_*int*_) was 4:1 for 1^st^ speed and 2:1 for 2^nd^ speed. The number of teeth on each gear was denoted by *T*_*1*_*, T*_*2*_*, T*_*3*_*, T*_*4*_*, T*_*5,*_ and* T*_*6*_. The input shaft (*N*_*i*_) from the motor ran at a maximum speed of 3882 rpm. The output speed ratio from the gearbox was:

1 $$\frac{{N}_{o}}{{N}_{i}}= \frac{{T}_{1}}{{T}_{2}}\times \frac{{T}_{5}}{{T}_{6}}$$

2 $$\frac{{N}_{i}}{{N}_{int}} =\frac{{T}_{2}}{{T}_{1}}$$

There was a fixed and constant centre distance between the two shafts of the gearbox. Therefore,

3 $${T}_{1} +{T}_{2} = {T}_{3}+ {T}_{4}={T}_{5} + {T}_{6}= constant$$

From the above equations, the number of teeth of different gears *T*_*1*_*, T*_*2*_*, T*_*3*_*, T*_*4*_*, T*_*5*,_ and *T*_*6*_ was 20, 80, 33, 67, 20, and 80, respectively.

A pair of helical gears transmitted a power (*P*) of 8000 W. The teeth were short in the diametral plane, with a pressure angle (*ϕ*) of 20° and a helix angle (*α*) of 30°. The motor shaft was connected to the pinion, which had a maximum rpm (*N*_*P*_) of 3882. Consider that the gears were made of cast steel and had an allowable static strength (*σ*_*OP*_) of 50 N/mm^2^. The pitch diameter of the gear (*D*_*G*_) was 200 mm, and the pitch diameter of the pinion (*D*_*P*_) was 50 mm were selected which had a surface endurance limit (*σ*) of 618 N/mm^2^. The pinion and the gears were made of cast steel, and the torque (*T*) transmitted by the pinion:

4 $$T =\frac{P \times 60}{{2 \pi \times N}_{P}}= 19.69 \mathrm{N}\mathrm{m}$$

Tangential tooth load on the pinion:

5 $$WT= \frac{T}{{D}_{P }/2}= 787.60 \mathrm{N}$$

Number of teeth on the pinion:

6 $${T}_{P}=\frac{{D}_{P }}{m}$$

Equivalent number of teeth for the pinion:

7 $${T}_{E} =\frac{{T}_{P}}{{\mathit{cos}}^{3}\alpha }=\frac{786.1}{m}$$

Tooth form factor for the pinion for 20° stub teeth:

8 $$Y_{P} ^{\prime} = 0.175 - \frac{0.841}{{T_{E } }} = 0.175 {-} 0.01m$$

Peripheral velocity:

9 $$v=\frac{{\pi D}_{P }{N}_{P }}{60} = 10.15 \mathrm{m}/\mathrm{s}$$

Velocity factor:

10 $${C}_{v}=\frac{15}{15 + v} = 0.59$$

A maximum face width (*b*) for the helical gears was taken as 12.5 times the module (*m*).

Tangential tooth load:

11 $${W}_{T} = ({\sigma}_{OP} \times {C}_{v}) b \times \pi \times m \times {Y}_{P}^{\prime}$$

From the above equation, module (*m*) and face width (*b*) were 2.1 mm and 26.2 mm.

Velocity ratio:

12 $$V.R =\frac{{D}_{G }}{{D}_{P }} = 4$$

Ratio factor:

13 $$Q=\frac{2 \times V.R.}{V.R. + 1} = 1.6$$

Normal pressure angle:

14 $${\phi}_{N}= {tan}^{-1} (tan \phi cos \alpha ) = 17.13^\circ$$

Both gears were made of the same material, i.e., cast steel; therefore, their effective stress on one gear (*E*_*P*_) and the other gear (*E*_*G*_) was 200,000 N/mm^2^.

Load stress factor:

15 $${K}_{L}=\frac{{\left(\sigma \right)}^{2}\mathit{sin}{\phi}_{N}}{1.4}\left(\frac{1}{{E}_{P}}+\frac{1}{{E}_{G}}\right)= 0.80 \mathrm{N}/{\mathrm{m}\mathrm{m}}^{2}$$

Maximum or limiting load for wear:

16 $${W}_{w} =\frac{{D}_{P} \times b \times Q{ \times K}_{L} }{{\mathit{cos}}^{2}\alpha }= 2240.00 \mathrm{N}$$

The design was considered satisfactory in terms of wear because the maximum wear load was greater than the tangential load on the tooth (*W*_*w*_ > *W*_*T*_).

**Fig. 3:**
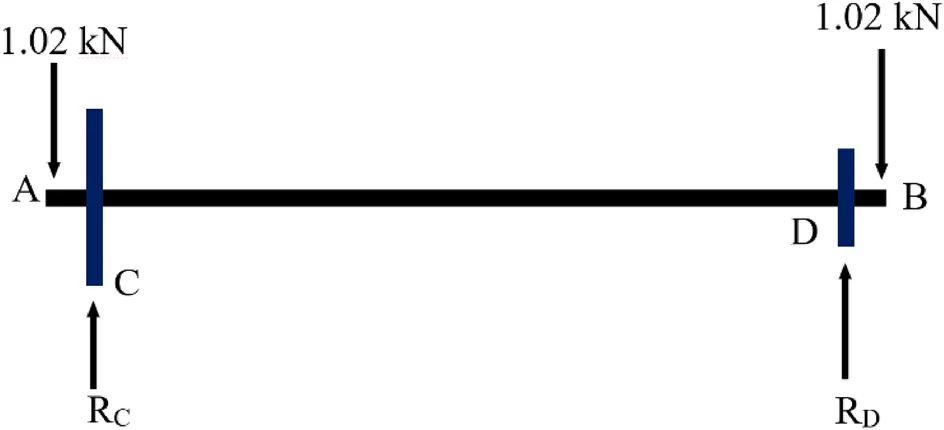
Bending moment at each axle shaft.

The maximum rotation of each rear axle (*N*_*s*_) of 478 rpm was used to transmit power (*P*_*s*_) of 4000 W. The axle was made of mild steel with an allowable shear stress (*τ*) of 42 N/mm^2^. The length of the axle was selected as 560 mm. An axle carried a load of 1.02 kN on both sides, acting at 40 mm from the brake drum. The bending moment at the axle shaft is shown in Fig. 3.

Bending Moment (B.M.) at A,

M_A_ = 0.

B.M. at C,

M_C_ = 1.02 × 10^3^ × 40 = 40.8 × 10^3^ N-mm.

B.M. at D,

M_D_ = 1.02 × 103 × 40 = 40.8 × 103 N-mm.

B.M. at B,

M_B_ = 0.

Torque transmitted by the shaft:

17 $${T}_{S} =\frac{{P}_{s} \times 60}{{2 \pi \times N}_{s}}= 79.95 \times {10}^{3} \mathrm{N}-\mathrm{m}\mathrm{m}$$

Diameter of the shaft:

18 $${d}^{3} =\frac{{T}_{s}\times 16}{\pi \times \tau } d = 21.33 \mathrm{m}\mathrm{m}$$

Therefore, the minimum diameter of each axle shaft required to transmit a power of 4000 W was 21.33 mm. The bearings and bushings were provided at the mounting points where the axle was supported. The axle was protected by enclosing the length of the axle in a housing. A conventional drum brake was installed at the end of the axle to control the speed of the wheels by pressing the hydraulic brake pedals^21^.

### Design of a chain drive

**Fig. 4:**
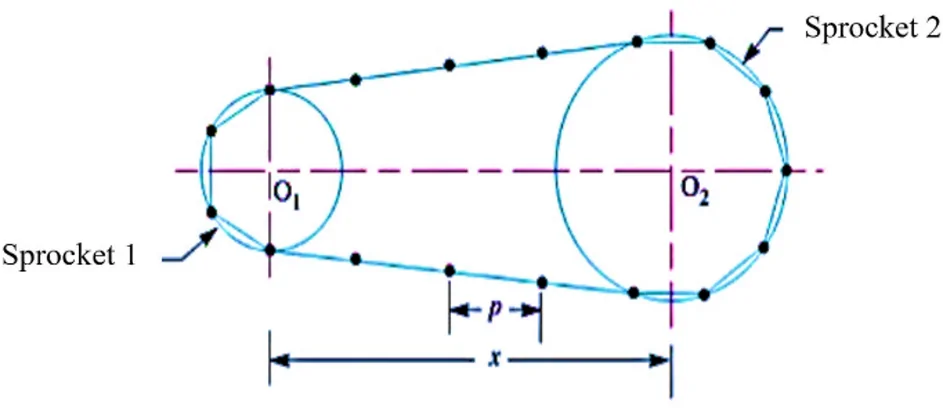
Chain drive.

Chain drives provide a positive, non-slip drive and can withstand higher torque transfer, offering greater durability and load capacity^22^. The chain drive system was selected to transfer power to a ground clearance e-vehicle as shown in Fig. 4. This power, originating from an 8000 W motor, is directed to the rear axle, where two sprockets are attached to both ends of the axle and another is linked to the wheels^20^.

The motor transmitted equal power to both chain drives through the gearbox at a rated power of 4000 W, with sprocket 1 having teeth (*T*_*S1*_) 15 that rotated (*N*_*S1*_) at 478 rpm, and sprocket 2 rotated (*N*_*S2*_) at 273 rpm. A chain drive was designed to transmit power from one sprocket to another.

Velocity ratio:

19 $${V}_{R} =\frac{{N}_{s1}}{{N}_{s2}}= 1.75$$

Number of teeth of sprocket 2:

20 $${T}_{S2}= {T}_{S1}\times \frac{{N}_{s1}}{{N}_{s2}} = 26$$

As per IS 2403:1991, load factor (*K*_*1*_), Lubrication factor (*K*_*2*_), and Rating factor (*K*_*3*_) were 1.25, 1.5, and 1, respectively.

Service factor:

21 $${K}_{s}= {K}_{1} \times {K}_{2} \times {K}_{3} = 1.8$$

Sprocket 1, with a maximum speed of 478 rpm and transmitted power of 4000 W, was paired with chain number 16 B according to the standard. This chain had pitch (*p*), roller diameter, width between inner plates, and transverse pitch was 25, 15, 17, and 31 mm, respectively. This chain had a breaking load (*W*_*B*_) of 42 kN.

Design power:

22 $${P}_{D} = rated \,\,power \times service\,\, factor ({K}_{s}) = 7200 W$$

Pitch circle of diameter of sprocket 1:

23 $${d}_{1} = p cosec (\frac{180}{{T}_{1}})= 120.17 \mathrm{m}\mathrm{m}$$

Pitch circle of diameter of sprocket 2:

24 $${d}_{2} = p cosec (\frac{180}{{T}_{2}})= 207.25 \mathrm{m}\mathrm{m}$$

Pitch line velocity:

25 $${V}_{1}=\frac{\Pi {d}_{1}{N}_{S1}}{60} = 3.00 \mathrm{m}/\mathrm{s}$$

Load on the chain:

26 $${L}_{C}= \frac{Rated \,\,power}{Pitch\,\, line \,\,velocity}= 1330 \mathrm{N}$$

Factor of safety:

27 $${F}_{s}=\frac{Breaking\,\, Load}{Pitch \,\,line \,\,velocity}= 31.57$$

As per the standard, the considered factor of safety was more than 10.33. Therefore, the chain drive did not break while transmitting power of 4000 W at 478 rpm. The centre distance between two sprockets (34 times of pitch) was selected.

Centre distance between sprockets:

28 *x = 34 × p = 850 mm*

Number of chain links:

29 $$K = \frac{{T}_{S1}-{T}_{S2}}{2}+ \frac{2 \times x }{p}+ (\frac{{T}_{S2}-{T}_{S1}}{2\pi }{)}^{2} \frac{p}{x}= 88.59 \sim 89$$

Length of the chain:

30 *L = K × p = 2225 mm*

The chain drive was designed with the pitch circle diameter of sprocket 1 and the pitch circle diameter of sprocket 2, the distance between the centres of the sprockets, and the length of the chain were 120.17 mm, 207.25 mm, 850 mm, and 2225 mm, respectively. The number of teeth on sprocket 2 and the number of chain links were selected to be 26 and 89, respectively. The dimensions of the chain-driven body were 80 × 330 × 1170 mm.

### Design calculations for motor requirement

The power needed to propel an e-vehicle had been calculated by combining the forces required to move it with the speed at which these forces had to be maintained^23^. The terrain was assumed to be soft, such as mud or sand, to maximize the rolling resistance force, with a coefficient of rolling resistance of *μ*_*R*_ = 0.2. The maximum allowable speed of the e-vehicle (*S*) was considered to be 3.33 m/s (or 12 km/h), the acceleration (*a*) was 0.33 m/s^2^, and the e-vehicle weight (*M*) was 1000 kg, including payload. These opposing forces were accounted for as follows:

Rolling resistance:

31 $${F}_{r}= {\mu}_{R} \times M \times g = 1960 N$$

Acceleration force:

32 $${F}_{a} = M \times a = 330 N$$

Total tractive force:

33 $${F}_{D}= {F}_{r}+ {F}_{a} = 2290 N$$

Power requirement to propel an e-vehicle:

34 $${P}_{V}={F}_{D} \times S = 7626 W$$

At the design stage, consider a maximum allowable speed of the e-vehicle of 3.33 m/s (or 12 km/h), acceleration (*a*) of 0.33 m/s^2^, and weight of the e-vehicle including payload (*M*) of 1000 kg. So, the power required to propel the e-vehicle was 7626 W.

### Permanent magnet synchronous motor and controller

The Permanent Magnet Synchronous Motor (PMSM) used in electric vehicles offers high efficiency, high torque at low speeds, a compact size, and an excellent power-to-weight ratio^24^. The Permanent Magnet Synchronous Motor (PMSM) was selected to propel the e-vehicle, which had 8000 W of power, high power density, and consistent torque delivery. The motor was operated at 72 V, had six phases, and could run at three different speeds. A controller was selected to govern the performance of the PMSM motor in a predetermined manner. The motor controller had an operating power range of 5000 to 8000 W, a working voltage of 72 V, and a current limit of 130 to 150 A. The motor and controller featured waterproofing, air-cooling design, fuse safety, and overload protection.

The pedal throttle system controlled the motor speed of the e-vehicle. The e-vehicle had three different speeds that could be adjusted through a speed switch, and it had a neutral, drive, and reverse (NDR) switch that allowed the e-vehicle to move forward, backward, and remain neutral. The e-vehicle was equipped with a brake switch that, when activated, automatically cut off the power to the motor, preventing it from running.

### Wiring diagram of an e-vehicle

**Fig. 5:**
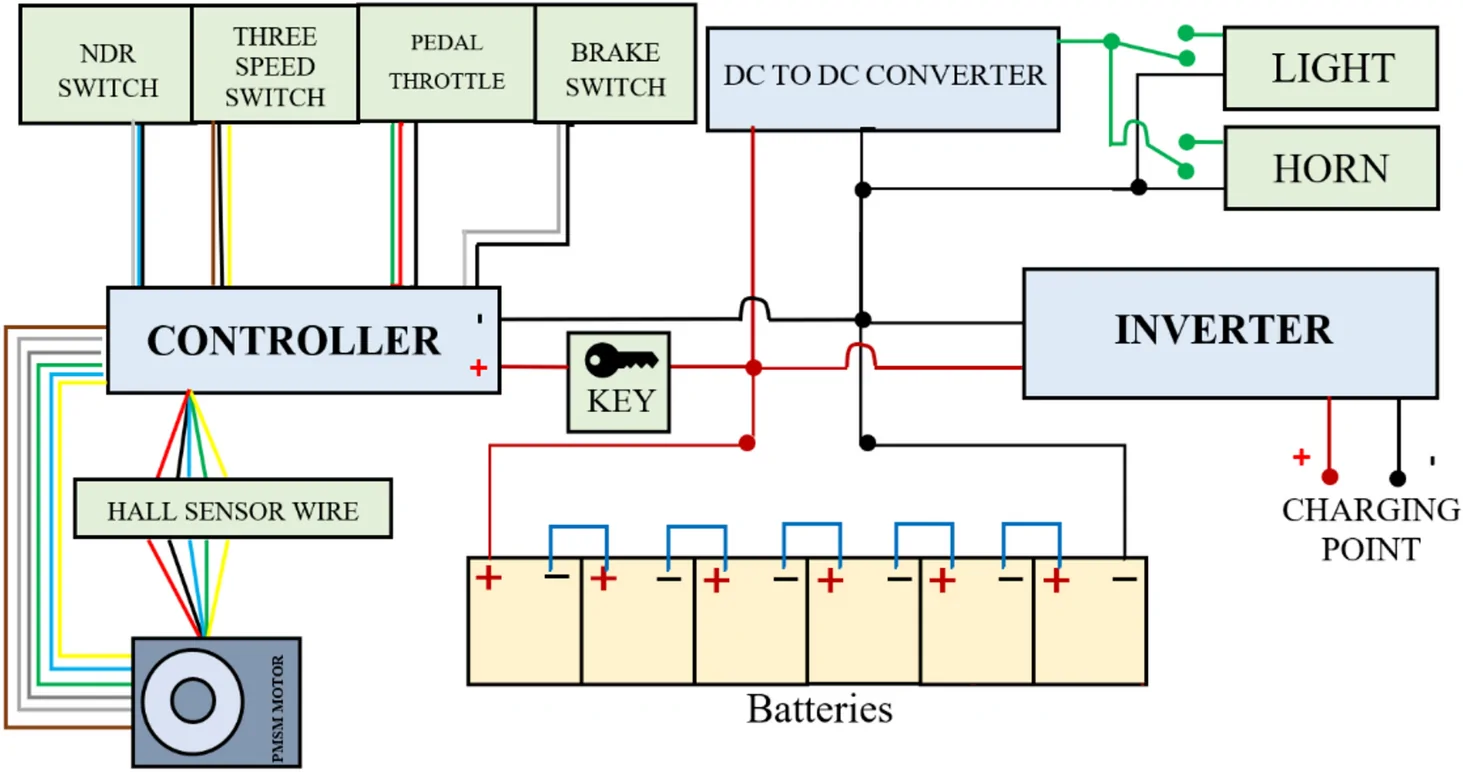
Circuit diagram of e-vehicle.

The components of e-vehicles, i.e., PMSM motor, controller, pedal throttle, Neutral-Drive-Reverse (NDR) switch, batteries, etc, were connected with a wire. The batteries were the power source of the vehicle, and the energy flow of the vehicle was in the form of electrical power^25^. Six lead-acid batteries, each rated at 12 V with a current capacity of 160 Ah, were connected in series, resulting in a total output voltage of 72 V while maintaining a capacity of 160 Ah. The circuit diagram of the e-vehicle is shown in Fig. 5.

### Power transmission

The PMSM motor had three-speed modes for forward drive and one-speed mode for reverse drive. The gearbox had two gear modes, namely gear 1 and gear 2. The rpm of the motor shaft, sprocket 1, and sprocket 2 were measured with the help of a tachometer. The speed of the power transmission system of the e-vehicle is shown in Table 2. The gear ratio between the motor shaft and sprocket 1 at gear 1 was 16:1, while at gear 2 it was 8:1. The gear ratio between the motor shaft and sprocket 2 at gear 1 was 28:1, while at gear 2 it was 14:1.

**Table 2:** Power transmission of e-vehicle from PMSM motor to wheel.

Motor speed mode	Motor shaft	Sprocket 1		Sprocket 2	
		At gear 1 (rpm)	At gear 2 (rpm)	At gear 1 (rpm)	At gear 2 (rpm)
1	2845	177	355	101	203
2	3289	205	411	117	234
3	3882	242	478	138	273
Reverse	1052	65	131	37	75

## Specifications of e-vehicle

**Fig. 6:**
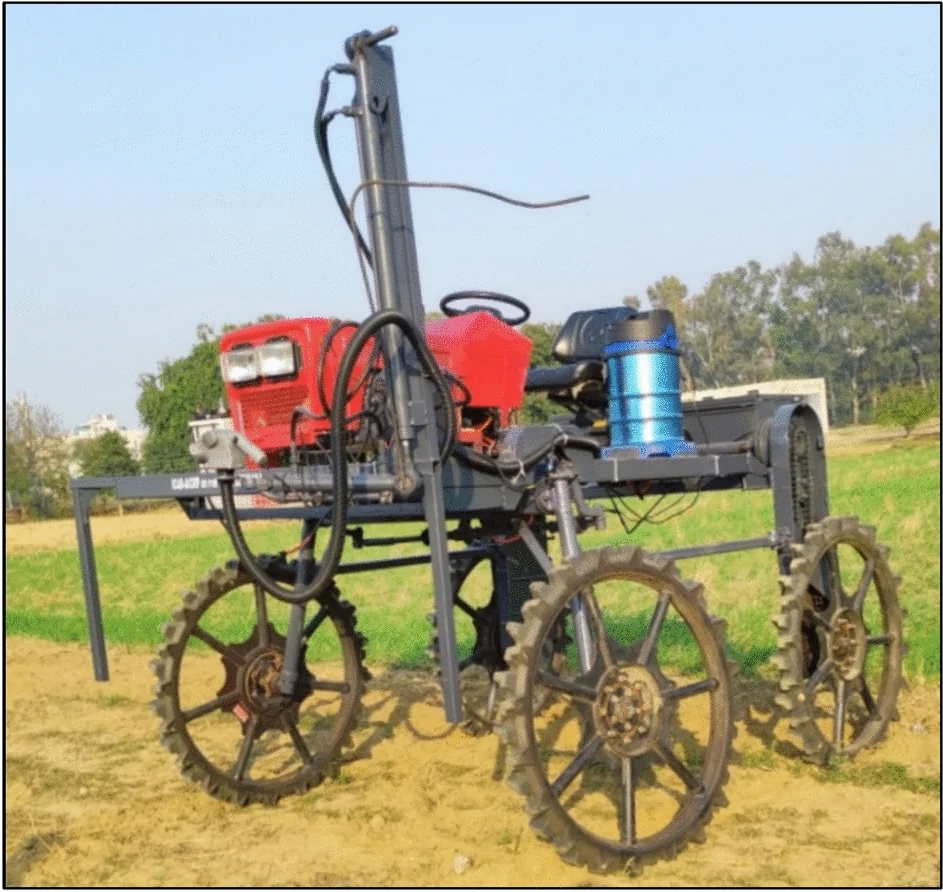
A view of the developed e-vehicle.

**Fig. 7:**
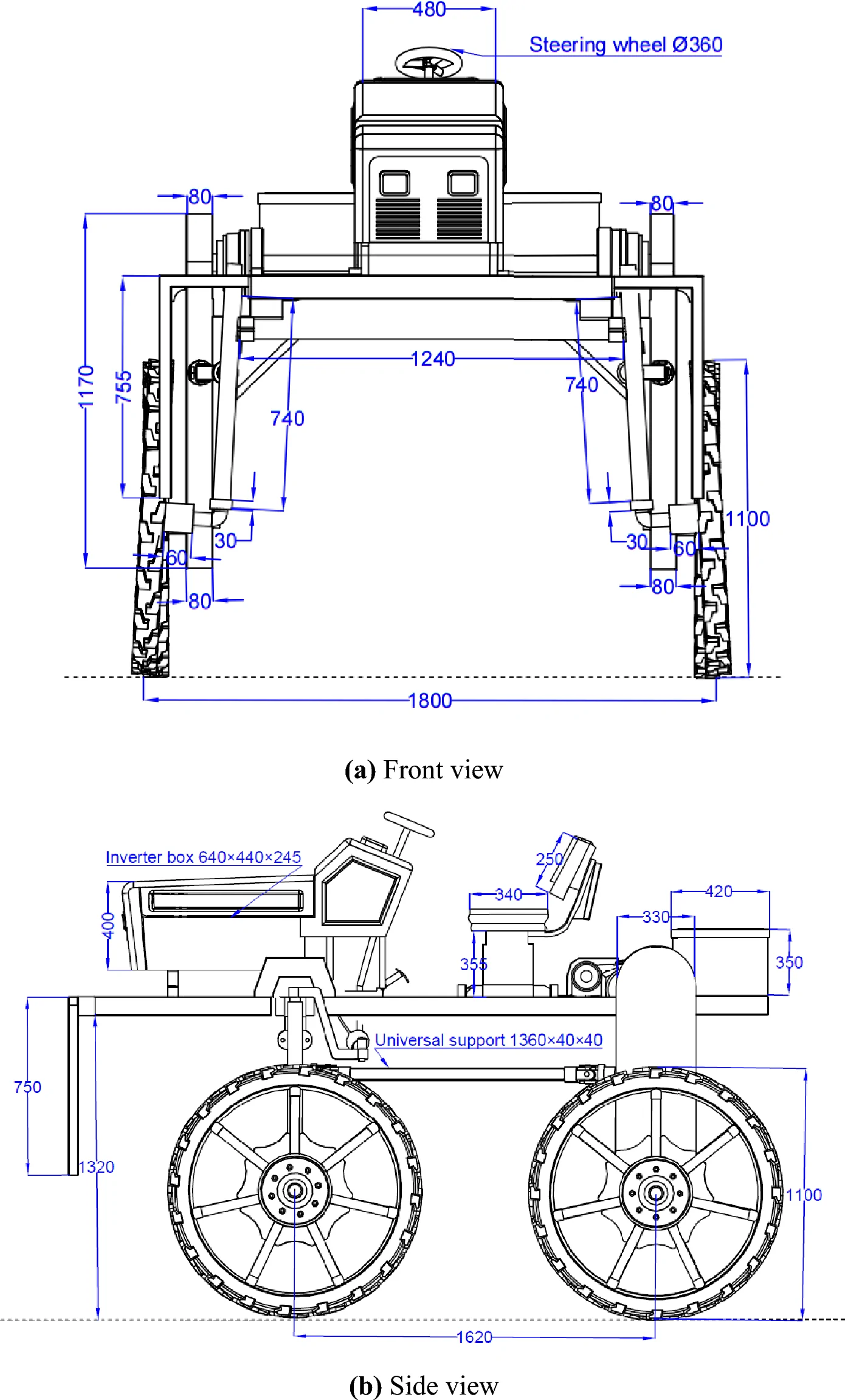

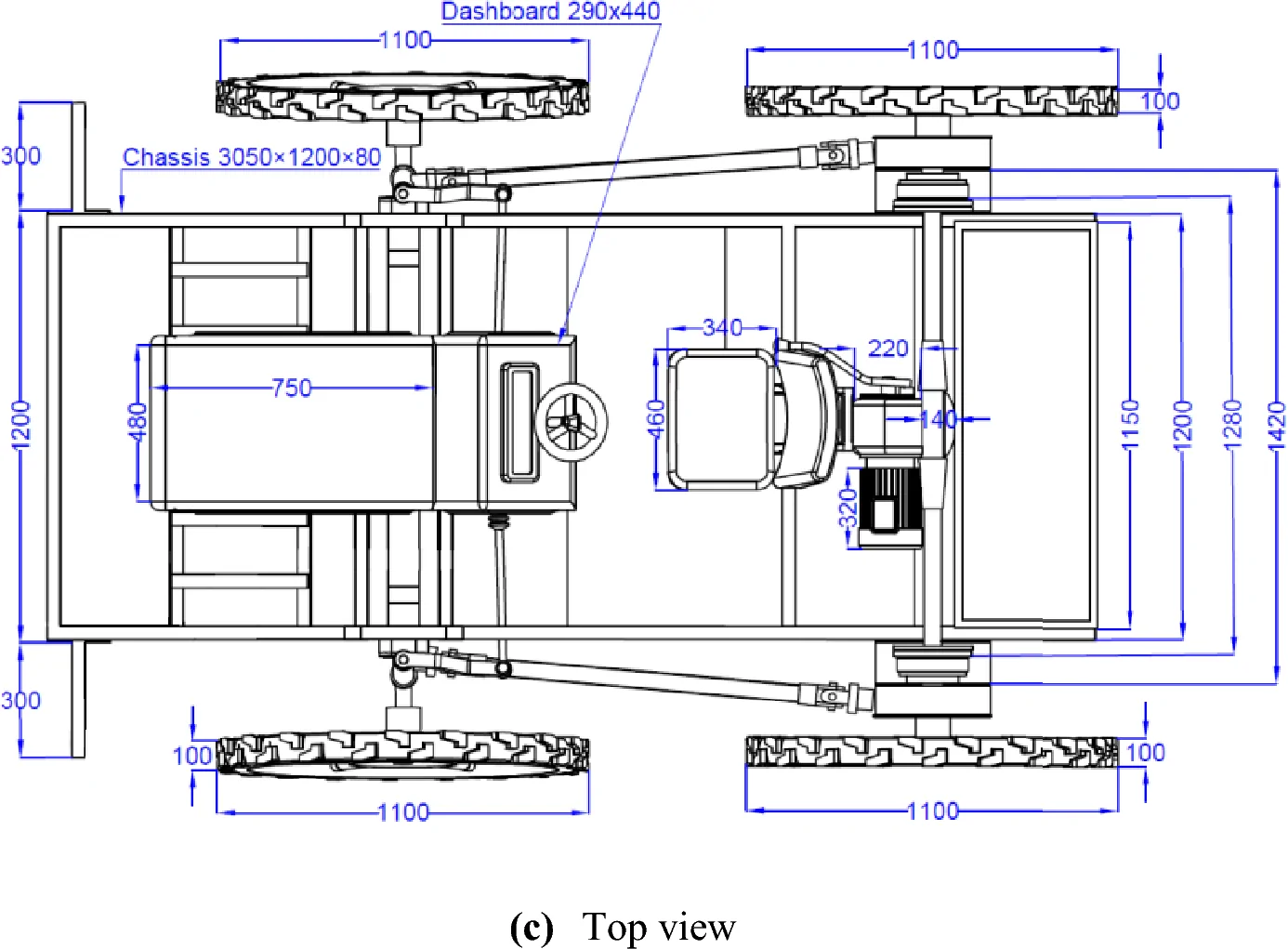
A 2D view of the developed e-vehicle with dimensions in mm **(a)** Front view, **(b)** side view, and **(c)** Top view.

The main specifications and view of the e-vehicle are given in Table 3 and Fig. 6. A 2D drafting of the e-vehicle was done with AutoCAD 2024 software as shown in Fig. 7.

**Table 3:** Specifications of the developed e-vehicle.

Sr. No.	Description	Specification
1.	Overall dimensions	
	Wheelbase, mm	1620
	Track width, mm	1800
	Ground clearance, mm	1320
	Weight, kg	830
2**.**	Non-pneumatic tire	
	Diameter, mm	1100
	Width, mm	100
3.	Chassis	
	Length, mm	3050
	Width, mm	1200
	Height, mm	80
4.	Chain drive body	
	Length, mm	80
	Width, mm	330
	Height, mm	1170

### Determination of centre of gravity (CG) and stability of e-vehicle

**Fig. 8:**
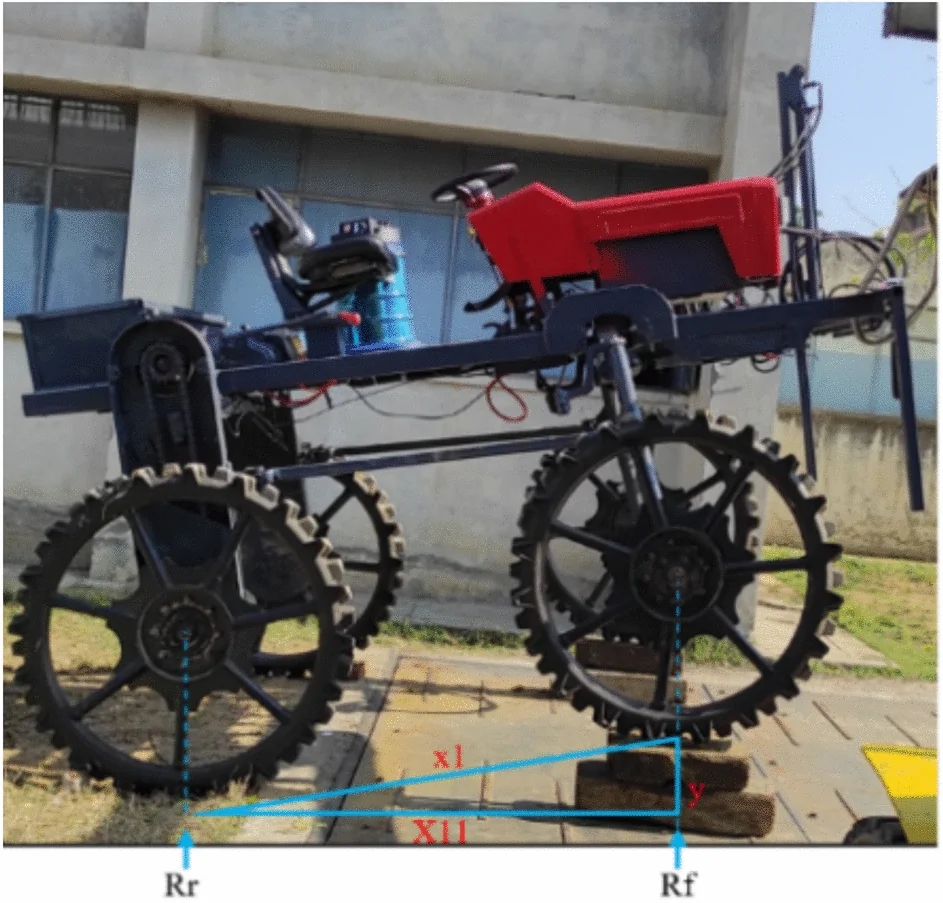
Uplift the front wheels with wooden blocks.

The centre of gravity of an e-vehicle represented the singular point where the total weight of the e-vehicle was regarded as concentrated. The centre of gravity of the e-vehicle was determined using a weighing method. The longitudinal position (x_2_) of the centre of gravity was determined by measuring the reactions on both the front (R_f_) and rear (R_r_) wheels. The total weight, as well as the reactions on the front and rear axles of the e-vehicle, were measured using a weighing bridge. The front axle of the e-vehicle was elevated in the air using a load lifter as shown in Fig. 8. The front axle of the e-vehicle was uplifted by 410 mm, and the reaction on the front axle (R_f1_) was subsequently rechecked^26, 27^.

**Fig. 9:**
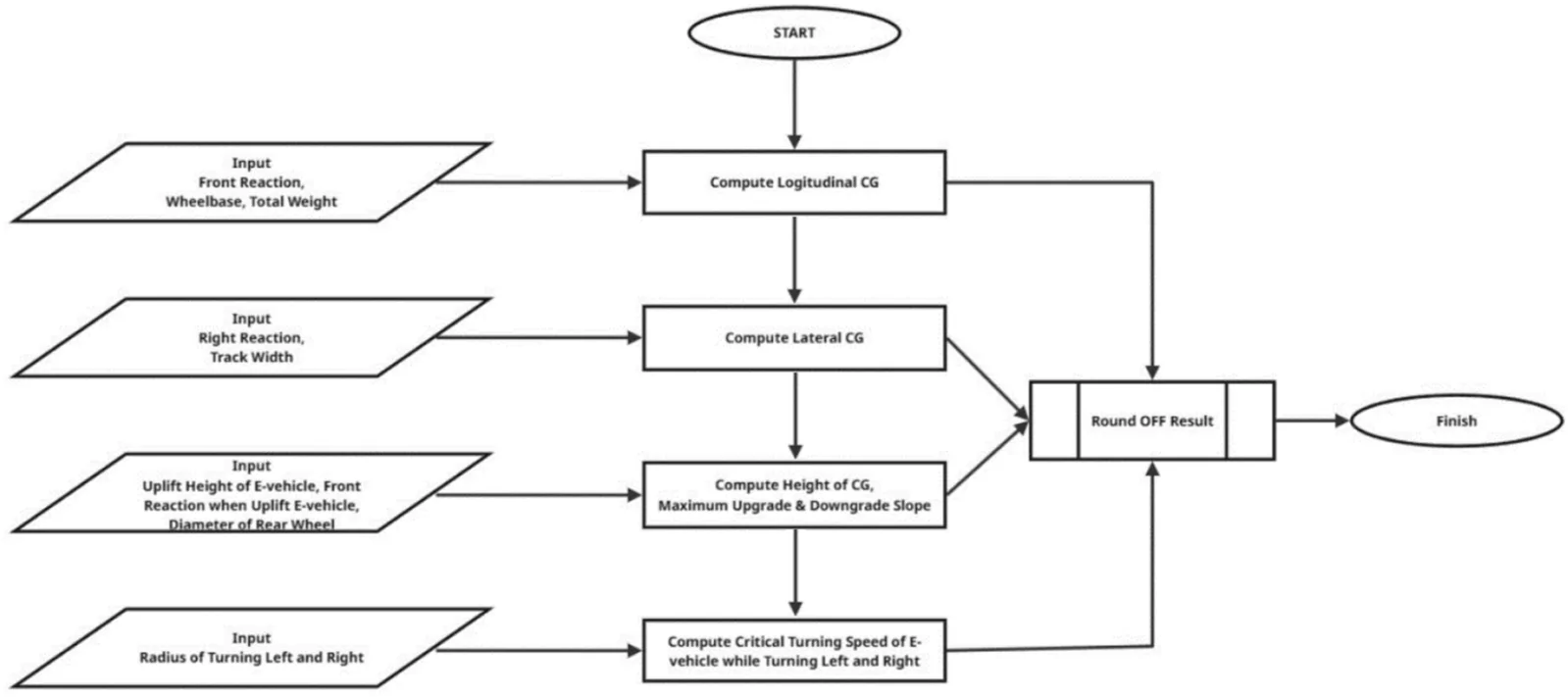
Steps to determine the stability parameters of the e-vehicle.

The e-vehicle was started at a marked point, the steering wheel was turned fully, and then it speed up, making circles in both left and right directions to assess stability while turning. The acceleration due to gravity (g = 9.8 m/s^2^) was considered. All formulas required to determine the centre of gravity were converted into an algorithm, into which all measured values were input, resulting in the outcome. The algorithm was developed in the Python language using the PyCharm integrated development environment (IDE) to determine the centre of gravity (CG) and the stability of the e-vehicle. The input values of the algorithm are given in Table 4. The steps of the algorithm to determine the stability parameters of the e-vehicle are shown in Fig. 9.

**Table 4:** Input values of the algorithm.

Sr. No.	Parameters	Value input
1.	Front reaction, kg	300
2.	Wheelbase, mm	1620
3.	Total weight of e-vehicle, kg	830
4.	Right reaction, kg	400
5.	Track width, mm	1800
6.	Uplift height of e-vehicle, mm	410
7.	Front reaction when uplift the e-vehicle, kg	228.5
8.	Diameter of rear wheel, mm	1100
9.	Radius of e-vehicle turn left, mm	5587
10.	Radius of e-vehicle turn right, mm	7308

### Moment of inertia of e-vehicle

The e-vehicle was mounted on a cradle for moment of inertia measurement, aligned with its transverse axis parallel to the cradle’s rotation axis and center of gravity in the vertical plane^28^. Secured by brakes and wooden blocks, oscillations were initiated, and the average duration for ten oscillations was recorded from five sets of readings.

The variables considered include the weight of the e-vehicle (W_v_ = 830 kg), cradle (W_c_ = 425 kg), and their combined weight (W_vc_ = 1255 kg). Key measurements included the distance from the pivot point to the centre of gravity (r), moment of inertia around the cradle’s rotation axis (I_₀_), and oscillation frequency (f_₀_). Additionally, the cradle’s moment of inertia (I_c_), the vehicle’s centre of gravity positions (X = 585.54 mm, Y = 1083.46 mm), vertical distance from platform to rotation axis (H_v_ = 2720 mm), and the distance between rotation axis and cradle’s centre of gravity (K_v_ = 2220 mm) were noted.

**Fig. 10:**
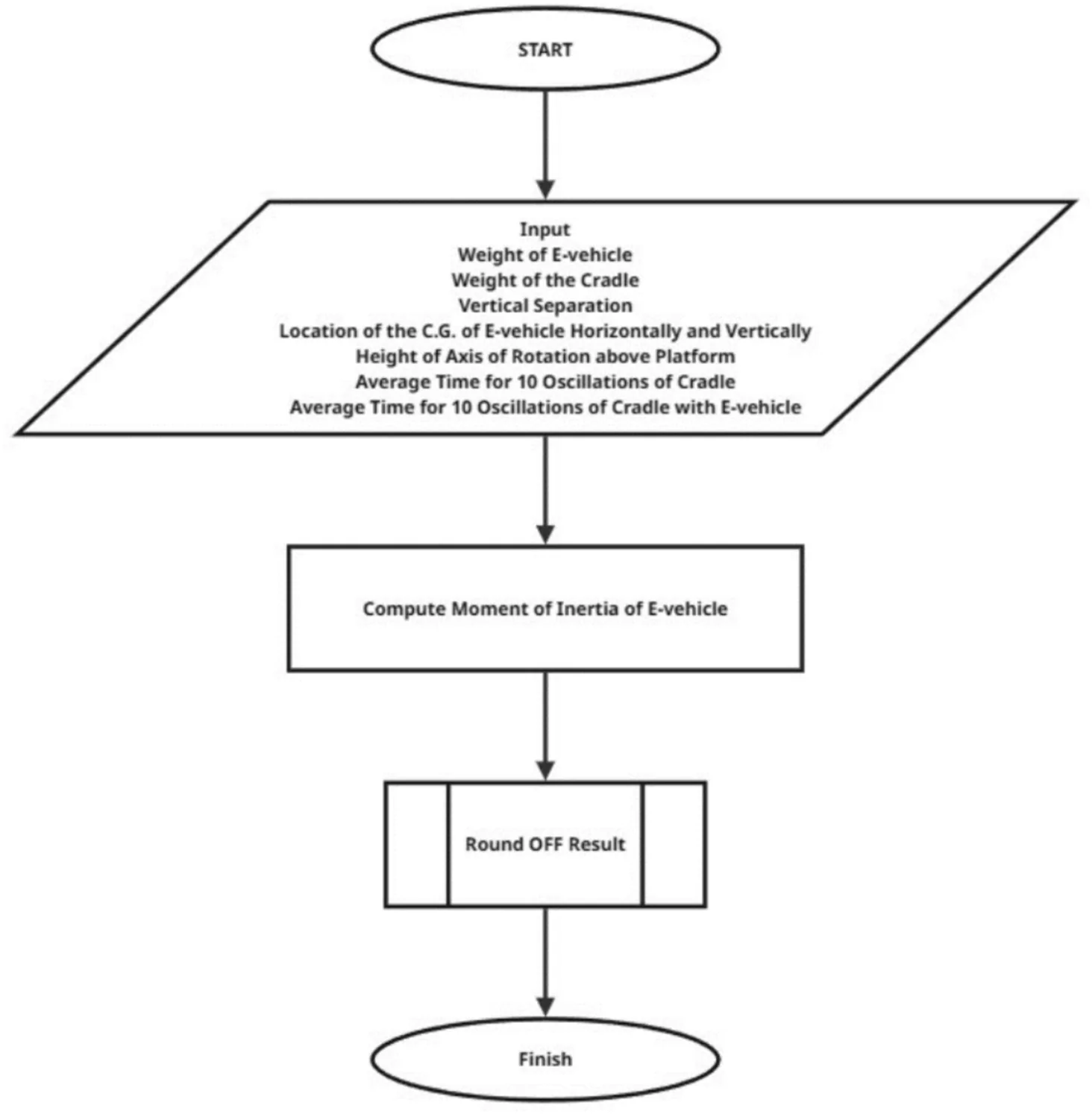
Steps to determine the moment of inertia of e-vehicle.

The times for 10 oscillations of the cradle were 32.08 s, 31.13 s, 32.23 s, 31.28 s, and 32.52 s. For the cradle and e-vehicle together, the times were 29.26 s, 30.18 s, 30.34 s, 30.20 s, and 29.37 s. The steps of the algorithm to determine the moment of inertia of the e-vehicle is shown in Fig. 10.

### Determination of travel speed of e-vehicle

The e-vehicle had covered a distance (D_c_) in a Time (T_t_)^29^. The travel speed of the e-vehicle was calculated with the following formula:

35 $$Travel \,\,Speed, TS =\frac{{D}_{c}}{{T}_{t}}$$

### Determination of wheel slip of e-vehicle

Electric motors enable the implementation of continuous wheel torque control, enhancing efficiency^30^. The distance ‘*A*’ represents the theoretical distance travelled by the e-vehicle for ten-wheel revolutions, calculated based on the wheel circumference. The actual distance travelled for the same number of wheel revolutions was measured experimentally and is denoted as ‘*B*’. Wheel slip (WS) was then calculated as:

36 $$Wheel \,\,Slip, WS =\frac{A-B}{A}\times 100$$

### Charging time and running time

The batteries of the e-vehicle were charged using an inverter. The charging time was recorded by noting the duration required to charge the batteries. After the batteries were fully charged, the running time of the batteries during field operation, as well as the running time of the e-vehicle, was recorded. The charging and running time were recorded in minutes (min).

## Results

### Stability of developed e-vehicle

The developed algorithms were run in PyCharm integrated development environment (IDE) to determine the centre of gravity (CG), stability of the e-vehicle, and moment of inertia of the e-vehicle. After running the algorithm to determine the CG and stability of the e-vehicle, required to fill the required input values as shown in Table 4. The resulting outcome is shown in Table 5.

**Table 5:** Output values of the algorithm.

Sr No.	Particular	Output
1	Longitudinal location of centre of gravity from the rear axle, mm	585.54
2	Lateral location of centre of gravity from the left side of rear wheel, mm	867.47
3	Height of centre of gravity from ground, mm	1083.46
4	Maximum upgrade slope at which e-vehicle remains stable, °	28.39
5	Maximum downgrade slope at which e-vehicle remains stable, °	43.67
6	Critical turning speed of e-vehicle while turning left, km/h	24.71
7	Critical turning speed of e-vehicle while turning right, km/h	27.26

Similarly, after running the algorithm, the moment of inertia of the e-vehicle about its centre of gravity was 51135.31 kg-mm-s^2^. This was measured along the transverse axis, which runs side to side and is perpendicular to the vehicle’s direction of travel. The moment of inertia indicated high rotational stability, making the e-vehicle more resistant to tipping and swaying.

### Operational parameters of e-vehicle

**Fig. 11:**
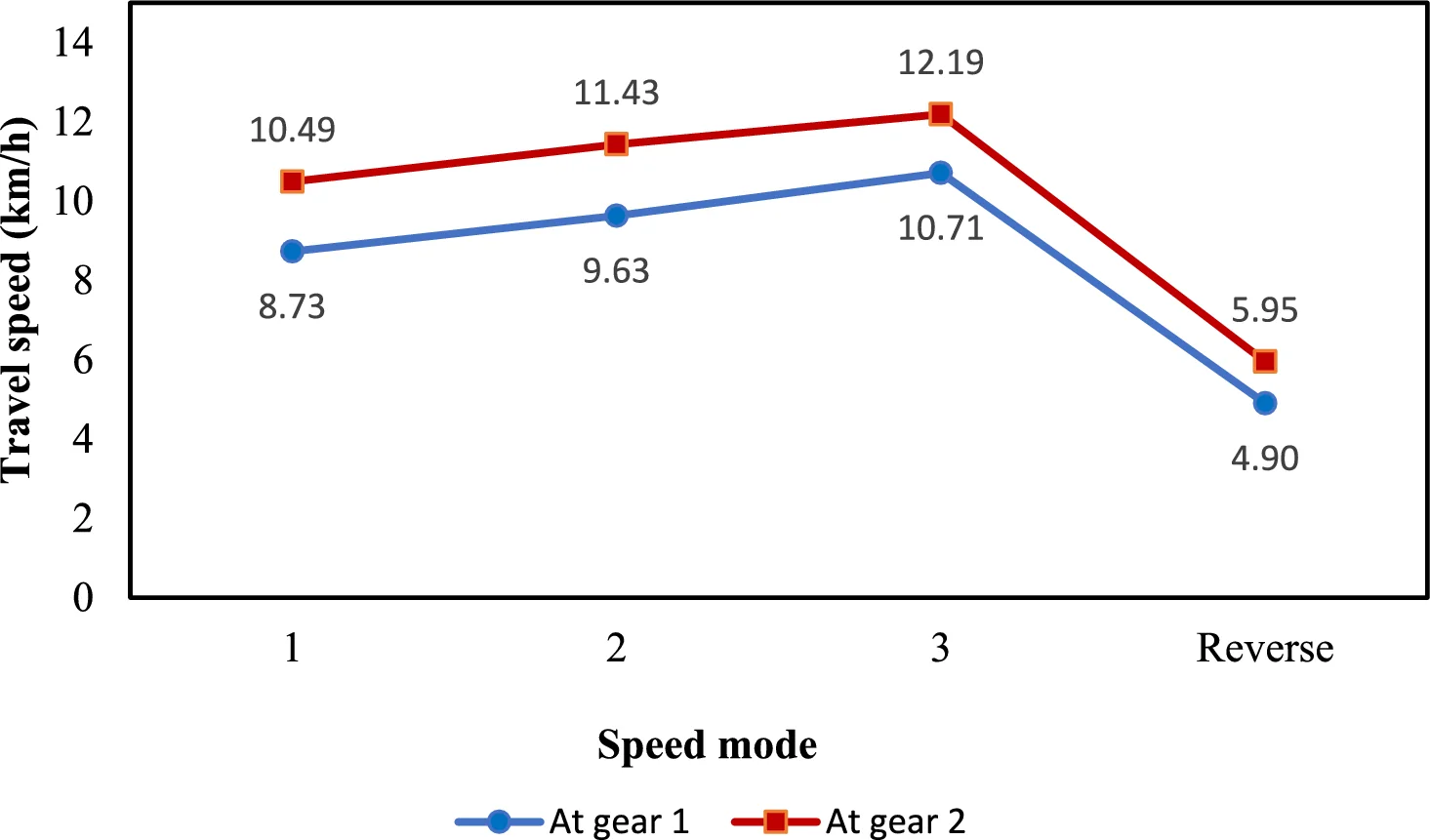
Travel speed of e-vehicle on the field.

The travel speed of the e-vehicle in the field depended on the PMSM motor speed modes (1, 2, and 3), which were controlled by a speed switch, while the reverse mode was controlled by the NDR switch. The travel speed was measured over multiple trials, as presented in Table 6, and the corresponding mean travel speed are shown in Fig. 11. The mean travel speed, obtained from multiple trials at different speed modes (1, 2, 3, and reverse), was 8.73, 9.63, 10.71, and 4.90 km/h at gear 1. At gear 2, the corresponding mean travel speeds were 10.49, 11.43, 12.19, and 5.95 km/h, respectively.

**Table 6:** Travel speed of e-vehicle on the field at different motor speed modes.

Gear	Trials	E-vehicle speed (km/h)			
		Mode 1	Mode 2	Mode 3	Reverse
Gear 1	1	8.62	9.53	10.51	4.79
	2	8.76	9.65	10.79	4.85
	3	8.81	9.71	10.83	5.06
	Mean	8.73	9.63	10.71	4.90
Gear 2	1	10.41	11.36	12.14	5.92
	2	10.44	11.41	12.15	5.94
	3	10.62	11.52	12.28	5.99
	Mean	10.49	11.43	12.19	5.95

**Fig. 12:**
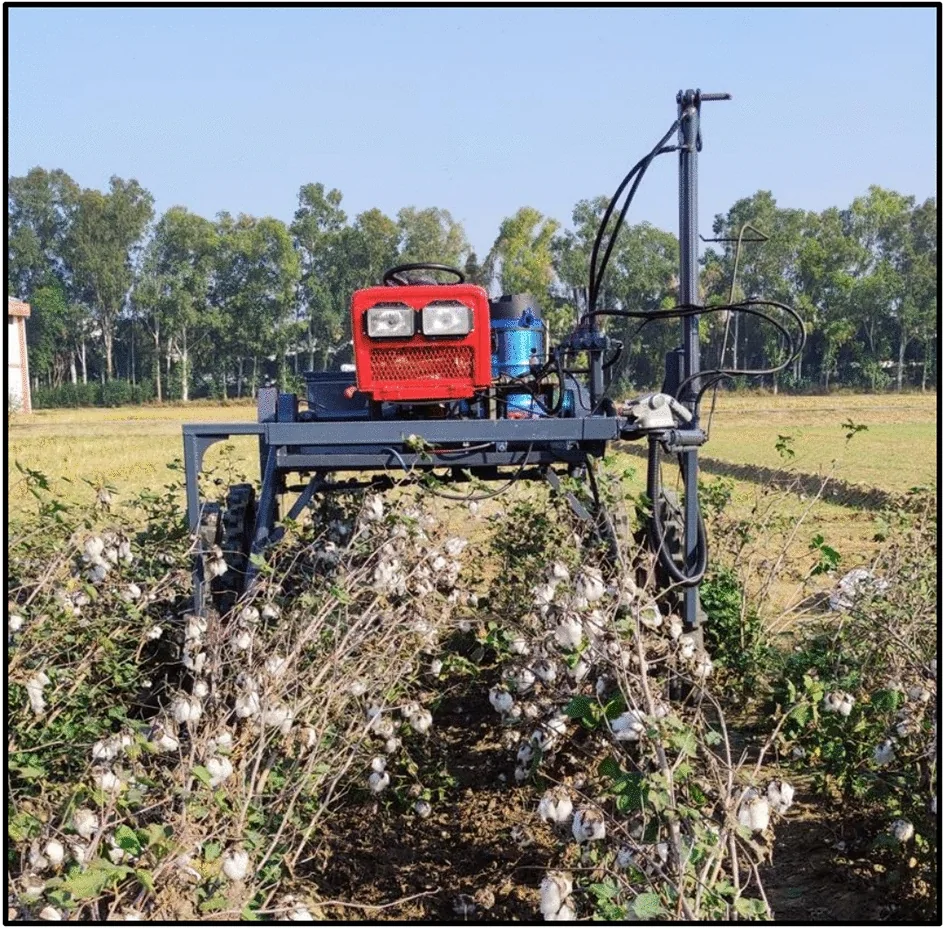
E-vehicle moves effectively through narrow cotton field rows with adequate ground clearance.

The e-vehicle had sufficient forward and reverse speed to run in the field. The e-vehicle had puncture-free non-pneumatic tires, measuring 1100 mm in length and 100 mm in width. These tires enabled the e-vehicle to propel through narrow cotton field rows with adequate ground clearance, as shown in Fig. 12. The operational parameters, including wheel slip, charging time, and running time of the e-vehicle, obtained from multiple experimental trials, are summarized in Table 7.

**Table 7:** Operational performance parameters of the e-vehicle under repeated trials.

Experimental Trials	Wheel Slip (%)	Charging Time (min)	Running Time (min)
1	3.10	596	266
2	3.95	599	271
3	4.62	605	273
Mean	3.89	600	270

During testing on a ploughed agricultural field, the e-vehicle exhibited wheel slip ranging from 3.10% to 4.62% across three experimental trials. The mean wheel slip was 3.89%. This indicates a relatively low level of wheel slip and ensures good traction and stability during field operations. The e-vehicle was equipped with a 72 V, 160 Ah battery, which exhibited a charging time ranging from 596 to 605 min across three experimental trials. The mean charging time was 600 minutes by using an inverter. After a full charge, the batteries provided sufficient power for the e-vehicle to operate continuously for 266 to 273 min at motor speed mode 1 and gear 1. The mean operating time under field conditions was 270 min.

## Discussion

The e-vehicle shows higher stability on a downgrade (43.67°) than on an upgrade (28.39°) because of the center of gravity shift. When moving uphill, the weight shifts toward the rear wheels, reducing the stabilizing effect on the front and making overturning more likely. In contrast, downhill, the weight moves forward, increasing normal force on the front wheels and improving resistance to tipping. Similar findings were reported by Roul and Singh^31^, where a high clearance vehicle remained stable up to 45.19°. The e-vehicle was evaluated on an agricultural field and is therefore safe against overturning. The e-vehicle might not overturn until the critical turning speed reaches 24.71 km/h for left turns and 27.26 km/h for right turns. The e-vehicle exhibited mean wheel slip 3.89% on a ploughed agricultural field, which is lower than the rear wheel slip reported for tractors using chisel plows (up to 18.78%) in the ANN-based study by Al-Dosary et al.^32^. The mean speed of the e-vehicle was 12.19 km/h in motor speed mode 1 at gear 2, which was lower than the critical speed,therefore, it remained stable against overturning in the field.

When the e-vehicle moved over crops with a row-to-row spacing of 900 mm in the field, two cotton rows came within the track width. The row-to-row spacing recommended by Punjab Agricultural University for cotton crops is 675 mm. When the e-vehicle was used for 675 mm row-to-row spacing, three cotton rows fit between the track width. The ground clearance of the e-vehicle was 1320 mm, and the maximum height of the cotton plant was 1302 mm observed for the Bt hybrid cotton variety RCH 846 BG II sown at 900 mm row spacing. Therefore, the crop was safe when the e-vehicle moved over it. The developed high-clearance e-vehicle serves as a dedicated mobility platform for robotic cotton picking. It provides stable field mobility while preventing crop damage by maintaining adequate ground clearance.

## Conclusions

In this study, an e-vehicle was designed and developed for a robotic cotton picker that was run over the cotton crop in the field with sufficient ground clearance. A permanent magnet synchronous motor of power 8000 W was selected for the e-vehicle. The location of the centre of gravity of the developed e-vehicle was 585.54 mm from the rear axle (longitudinal), 867.47 mm from the rear left side (lateral), and 1083.46 mm from the ground (height). The maximum upgrade and downgrade slopes at which the e-vehicle remains stable were 28.39 and 43.67°, respectively. The critical turning speed of the e-vehicle while turning left and right was 24.71 and 27.26 km/h, respectively. The moment of inertia of the e-vehicle about its centre of gravity was 51135.31 kg-mm-s^2^, measured along the transverse axis that was perpendicular to the direction of the vehicle’s travel. The mean travel speed, obtained from multiple trials at different speed modes (1, 2, 3, and reverse), was 8.73, 9.63, 10.71, and 4.90 km/h at gear 1, while at gear 2 it was 10.49, 11.43, 12.19, and 5.95 km/h, respectively. The e-vehicle demonstrated a mean wheel slip of 3.89% on a ploughed agricultural field. The batteries required a mean charging time of 600 minutes and once fully charged, supported a mean operating duration of 270 minutes under field conditions. The e-vehicle demonstrated stable and safe operation while traversing slopes, executing turns, and navigating within dense cotton fields. Its low wheel slip, adequate travel speed, and satisfactory operating time indicate reliable traction and confirm its suitability for agricultural applications.

### Future perspectives and study limitations

This study presents the design and initial validation of a high-clearance e-vehicle as a mobility platform for robotic cotton picking; however, certain limitations should be acknowledged. The experimental evaluation was conducted under specific field conditions, and broader testing across diverse soil types, crop conditions, and payload variations would further strengthen the findings. Future research should focus on enabling autonomous operation, adapting the vehicle for use with different crops to enhance farming efficiency, and exploring its commercialization potential to deliver advanced automated solutions for modern agriculture.

## Data Availability

The datasets used during this study can be provided upon reasonable request from the corresponding author at the email: rahuly7234@gmail.com.

## Appendix 1 An algorithm to determine the centre of gravity (CG) of the e-vehicle is given below:

#Longitudinal location of the centre of gravity from the rear axle

R_f_ = float(input("Front reaction, kg = ")).

x_1_ = float(input("Wheelbase, mm = ")).

W_T_ = float(input("Total weight of e-vehicle, kg = ")).

x_2_ = (R_f_ * x_1_) / W_T._

#Lateral location of the centre of gravity from the rear left side:

R_R_ = float(input("Right reaction, kg = ")).

T_w_ = float(input("Track width, mm = ")).

x_L_ = (R_R_ * T_w_) / W_T._

#Height of the centre of gravity above the ground.

y = float(input("Uplift height of e-vehicle, mm = ")).

R_f1_ = float(input("Front reaction when uplift the e-vehicle, kg = ")).

d_r_ = float(input("Diameter of rear wheel, mm = ")).

from math import asin, cos, tan, atan, degrees, radians, sqrt.

β_1_ = degrees(asin(y / x_1_)).

x_11_ = x1 * cos(radians(β_1_)).

x_21_ = (R_f1_ * x_11_) / W_T._

y_3_ = (x_2_ - (x_21_ / cos(radians(β_1_)))) / tan(radians(β_1_)).

H = (d_r_ / 2) + y_3._

# Longitudinal stability when the e-vehicle moves uphill:

θ_1_ = degrees(atan(x_2_ / H)).

# Longitudinal stability when the e-vehicle moves downhill:

x = x_1_-x_2._

θ_2_ = degrees(atan(x / H)).

# Stability of e-vehicle while turning:

T_L_ = float(input("Radius of e-vehicle turn left , mm = ")).

T_R_ = float(input("Radius of e-vehicle turn right , mm = ")).

g = 9.8 # m/s^2.^

S_L_ = 3.6 * sqrt(g * (T_L_/1000) * ( (T_w_-x_L_) / H)).

S_R_ = 3.6 * sqrt(g * (T_R_/1000) * ( x_L_ / H)).

# Round the results to two decimal places.

x_2_ = round(x_2_, 2).

x_L_ = round(x_L_, 2).

H = round(H, 2).

θ_1_ = round(θ_1_, 2).

θ_2_ = round(θ_2_, 2).

S_L_ = round(S_L_, 2).

S_R_ = round(S_R_, 2).

#Result print.

print("Longitudinal location of centre of gravity from the rear axle, mm = ", x_2_).

print("Lateral location of centre of gravity from the left side of rear wheel, mm = ", x_L_).

print("Height of centre of gravity from ground, mm = ", H).

print("Maximum upgrade slope at which e-vehicle remains stable, ° = ", θ_1_).

print("Maximum downgrade slope at which the e-vehicle remains stable, ° = ", θ_2_).

print("Critical turning speed of e-vehicle while turning left, km/h = ", S_L_).

print("Critical turning speed of e-vehicle while turning right, km/h = ", S_R_).

## Appendix 2 An algorithm to determine the moment of inertia of the e-vehicle is given below:

Import math.

# Input values.

W_v_ = float(input("Weight of e-vehicle, kg = ")).

W_c_ = float(input("Weight of the cradle, kg = ")).

K_v_ = float(input("Vertical separation between the axis of rotation and the cradle’s centre of gravity, mm = ")).

X = float(input("Location of the C.G. of e-vehicle horizontally from the rear wheel, mm = ")).

Y = float(input("Location of the C.G. of e-vehicle vertically from the ground level, mm = ")).

H_v_ = float(input("Height of axis of rotation above platform, mm = ")).

T_c_ = float(input("Average time for 10 oscillations of cradle, sec = ")).

T_cv_ = float(input("Average time for 10 oscillations of cradle with e-vehicle, sec = ")).

W_vc_ = W_v_ + W_c._

f_1_ = 10 / T_c._

f_o_ = 10 / T_cv._

I_c_ = (W_c_ * K_v_) / (4 * math.pi * math.pi * f_1_ * f_1_).

r = ((K_v_ * W_c_) + ((H_v_ - Y) * W_v_)) / (W_c_ + W_v_).

I_o_ = (W_vc_ * r) / (4 * math.pi * math.pi * f_o_ * f_o_).

g = 9.8 # m/s^2.^

I_CG_ = I_o_ - I_c_ - (W_v_ / g) * (H_v_ - Y) * (H_v_ - Y).

# Round the results to two decimal places.

I_CG_ = round(I_CG_, 2).

# Result.

print("Moment of inertia of e-vehicle about its C.G., kg-mm-s^2^ = ", I_CG_).
